# Correction: Fetal echocardiographic parameters in pregnancies complicated by diabetes: a case control study

**DOI:** 10.1186/s12884-024-07016-7

**Published:** 2025-01-09

**Authors:** Amal Darwish, Maged Abdel-Raouf, Rasha Kamel, Emad Salah, Mai Salah, Ahmed Okasha

**Affiliations:** 1 Department of Obstetrics and Gynecology, Faculty of Medicine, Kasr El Aini Hospital, Cairo University, Cairo, Egypt; 2 High Risk Pregnancy Unit, Department of Obstetrics and Gynecology, Faculty of Medicine, Kasr El Aini Hospital, Cairo University, Cairo, Egypt; 3 Maternal Fetal Medicine Unit, Department of Obstetrics and Gynecology, Kasr El Aini Hospital, Cairo University, Cairo, Egypt; 4 Department of Obstetrics and Gynecology, Embaba General Hospital, Egyptian Ministry of Health and Population, Giza, Egypt; 5 Department of Reproductive Health and Family Planning, Medical Research Institute, National Research Centre, Cairo, Egypt

**Correction: BMC Pregnancy Childbirth 22, 650 (2022)**

10.1186/s12884-022-04969-5

Following publication of the original article [1], the authors reported an error in Supplementary file. The author uploaded the wrong e-file.

The correct Supplementary file has been included in this correction, and the original article has been corrected.

## Electronic supplementary material

Below is the link to the electronic supplementary material.

Supplementary Material 1
